# Evaluating the safety and efficacy of cryopreserved ovarian tissue transplantation in leukemia patients with different bone marrow remission status using xenotransplantation

**DOI:** 10.3389/fendo.2024.1364316

**Published:** 2024-03-25

**Authors:** Yanqiu Li, Xiangyan Ruan, Muqing Gu, Juan Du, Fengyu Jin, Jiaojiao Cheng, Yanglu Li, Lingling Jiang, Zecheng Wang, Yu Yang, Mingzhen Zhang, Alfred O. Mueck

**Affiliations:** ^1^ Department of Gynecological Endocrinology, Beijing Obstetrics and Gynecology Hospital, Capital Medical University, Beijing Maternal and Child Health Care Hospital, Beijing, China; ^2^ Department for Women’s Health, University Women’s Hospital and Research Center for Women’s Health, University of Tuebingen, Tuebingen, Germany

**Keywords:** fertility preservation, ovarian tissue cryopreservation, ovarian tissue transplantation, leukemia, malignancy, xenotransplantation

## Abstract

**Background:**

Leukemia patients undergoing cryopreserved ovarian tissue transplantation (OTT) may carry a high risk of disease induction. Measurable residual disease (MRD) in bone marrow is linked to an elevated risk of relapse. It is controversial whether leukemia patients must be allowed to achieve measurable residual disease negative (MRD-negative) status instead of measurable residual disease positive (MRD-positive) status before ovarian tissue cryopreservation (OTC).

**Objective:**

To explore the safety and efficacy of OTT in acute leukemia patients with different MRD status by using xenotransplantation.

**Method:**

Cryopreserved ovarian tissue from 19 leukemia patients was thawed and xenotransplanted to ovariectomized BALB/C nude mice (n=36). The mice were divided into 2 groups based on the patient’s MRD status before OTC: MRD-negative group (n=18) and MRD-positive group (n=18), additionally, a control group consisted of ovariectomized mice (n=9). Body weight was measured weekly and mortality, emaciation, and other abnormalities were recorded. Twenty-six weeks post-surgery, livers, spleens, uteruses, and ovarian grafts were removed for macroscopic and histological examinations to evaluate the efficacy of xenotransplantation and assess malignant cell contamination in mice.

**Results:**

Follicle growth was visible in the ovarian grafts of the MRD-negative and MRD-positive groups. Compared with the ovariectomized group, a significant decrease in body weight (*p*<0.01) was noted, the uterine volume was notably larger, estradiol (E2) levels were significantly higher (*p*<0.01), and follicle-stimulating hormone (FSH) levels were significantly lower (*p*<0.001) in the other two groups. Mice in the MRD-positive group showed a significantly higher incidence of death (*p*<0.001) and emaciation (*p*<0.01), compared to the MRD-negative group. Histological observation revealed the presence of malignant cells in the grafts, livers, and spleens of 3 mice in the MRD-positive group. No abnormalities were observed in the mice from the MRD-negative group in both macroscopic and histological observations except one mouse was sacrificed for ascites unrelated to leukemia relapse.

**Conclusion:**

For leukemia patients having ovarian tissue preserved in the first and only centralized human ovarian tissue cryobank in China, immunodeficient mice xenotransplantation can be a method to evaluate the safety and efficacy of OTT; the risk of malignant cell reimplantation due to OTT is higher in leukemia patients with MRD-positive status than those with MRD-negative status before OTC.

## 1 Introduction

With the progression of therapeutic advancements, there has been a rapid decrease in the mortality rate of leukemia patients, accompanied by significant improvements in prognosis and an increase in life expectancy (1). However, the myeloablative conditioning undertaken before hematopoietic stem cell transplantation (HSCT) presents a potential risk of premature ovarian insufficiency (POI) (2, 3). For pediatric or young female patients with hematological diseases eligible for hematopoietic stem cell transplantation, fertility preservation is strongly advised, particularly OTC, before gonadotoxic therapy (4, 5). OTC stands out as the only fertility preservation method for children and women of reproductive age who are unable to delay gonadotoxic therapy (6).

However, a critical consideration is that leukemia can lead to malignant cells appearing throughout the entire circulatory system, which naturally involves the ovaries (7). To date, no measure eliminates the possibility of reimplanting malignant cells in ovarian tissue. If leukemia patients undergo OTT, there will be a risk of disease relapse (8). Due to the lack of studies on the safety of OTT, many experts warn leukemia patients to be cautious about this technique (9).

The autopsy in leukemia patients indicates the ovarian metastasis rate is 8.4% (10), but when the ovarian tissue is harvested for fertility preservation, the contamination rate with malignant cells reaches 24% (11). At the same time, OTC remains the primary fertility preservation strategy of female leukemia patients (12). Rigorous safety assays should be undertaken before OTT, and long-term follow-up evaluations are essential after it (13, 14).

A small number of leukemia cells remain in the bone marrow of a leukemia patient who has achieved a hematological complete remission (CR, defined as <5% of primitive cells detected by light microscopy in bone marrow) in bone marrow after treatment. Measurable residual disease (MRD) is defined as a low level of residual leukemia that is not detectable by morphological assessment alone. Thus, the disease state of leukemia patients can be subdivided into two categories: MRD-negative and MRD-positive. As the sensitivity of the MRD assays can be as high as 10^-2^ to 10^-6^, patients who have achieved CR by bone marrow morphology assessment alone may still have a large number of malignant cells in their bone marrow (15). Several studies have shown that MRD assays can be used for efficacy assessment and early warning of leukemia recurrence (16, 17). Studies in both adult and pediatric leukemia patients have found that MRD-positive in bone marrow is linked to an elevated risk of relapse and a shortened overall survival (18–20). MRD-negative patients had a significantly lower risk of recurrence and better prognosis than MRD-positive patients.

In previous studies on the safety of OTC in leukemia patients, it was found that OTT was safer for patients who achieved CR before OTC (12, 21). However, few studies are comparing in more detail whether there are differences in the safety of OTT in patients with different MRD status. Most patients have achieved CR with chemotherapy by the time OTC is planned, but MRD status and the prognosis of the patients vary considerably. There are several patients with recurrent MRD positivity after chemotherapy who are in urgent need of HSCT. Therefore, it is controversial whether patients must be allowed to achieve MRD-negative before OTC. Whether patients who test MRD-negative before OTC can feel more at ease with their decision, and whether they can shift from the perception that OTC in leukemia patients entails greater risk, remain controversial. These questions are matters of concern for both patients and healthcare professionals.

In this study, we successfully used xenotransplantation of human cryopreserved ovarian tissue from leukemia patients to investigate the safety and efficacy of OTT, and for the first time, MRD-negative leukemia patients undergoing OTC were used as independent research subjects. Assessment of malignant cell contamination was performed by observing differences in survival, macroscopic, and microscopic histology in xenograft models between MRD-negative and MRD-positive leukemia patients. This study furnished a theoretical basis for the clinical utilization of OTT in leukemia patients with varying MRD states.

## 2 Method

### 2.1 Patients

This study involved 19 female patients from 3 to 28 years old. **Table 1** showed that all of the patients were suffering from acute leukemia. Among them, 11 patients were diagnosed with acute myeloid leukemia, and 8 patients were diagnosed with acute lymphoblastic leukemia.11 patients were in MRD-positive status and 8 patients were in MRD-negative status. The MRD-positive status includes the following two situations. The first is when bone marrow morphology is in a state of non-remission or partial remission. The second is when bone marrow morphology has achieved CR, but at least one molecular MRD detection result is positive, using techniques such as multicolor flow cytometry (MFC), next-generation sequencing (NGS), or real-time quantitative polymerase chain reaction (RQ-PCR). All of the patients underwent OTC for fertility preservation at Beijing Obstetrics and Gynecology Hospital, Capital Medical University. Ovarian tissue was collected from clinical sub-centers and transferred to the ovarian tissue cryobank. All participants signed written informed consent forms, and for all patients under the age of 18, written consent was obtained from their parents. Consent was granted to allocate no more than 10% of the ovarian tissue for research purposes. The research involving human participants (tissue obtained from 19 leukemia patients) was approved by the Ethics Committee of Beijing Obstetrics and Gynecology Hospital, Capital Medical University (ethics NO: 2020-KY-007-01; date: April 20 2020.).

**Table 1:** Characteristics of the 19 patients who underwent OTC.

Patient number	Diagnosis	Age at OTC(years)	MRD status	Viable follicle count in fresh tissues (2 mm)	Viable follicle count in thawed tissues (2mm)
1	AML	25	positive	19	18
2	AML	20	positive	49	51
3	B-ALL	13	positive	867	872
4	B-ALL	28	positive	64	60
5	AML	14	positive	158	150
6	AML	14	positive	28	29
7	AML	21	positive	55	52
8	B-ALL	5	positive	925	935
9	B-ALL	6	positive	367	382
10	B-ALL	17	positive	204	210
11	AML	3	positive	163	172
12	AML	27	negative	12	10
13	AML	26	negative	9	8
14	AML	5	negative	39	35
15	B-ALL	27	negative	37	35
16	AML	22	negative	35	40
17	AML	27	negative	23	26
18	B-ALL	15	negative	78	75
19	B-ALL	25	negative	39	35

Patients’ age at OTC: 17.89 ± 8.24 years (range: 3–28 years old); AML,acute myeloid leukemia; ALL, acute lymphoblastic leukemia. The ovarian tissue used for viable follicle count is circular with a diameter of 2mm (the area is 3.14mm^3^).

### 2.2 Ovarian tissue cryopreservation and thawing

The ovarian sample was prepared in a sterile laminar flow cabinet at 4°C using Custodiol^®^ HTK solution (Dr. Franz Köhler Chemie GmbH). Subsequently, the ovarian tissue was stripped of the medulla, leaving only the ovarian cortex intact. The cortex was cut into thin slices measuring approximately 4mm*8mm*1mm each for future autotransplantation. The processed ovarian cortex slices were placed into 1.8ml standard cryotubes (NuncTM cryotubes, Thermo Fisher Scientific, Roskilde, Denmark) containing 1.7ml cryoprotectant solution. The cryoprotectants contain Leibovitz’s L-15 GlutaMAX medium (Gibco, USA) supplemented with 10% CryoSure-DMSO (WAK-Chemie Medical GmbH) and 1% human serum albumin (IrvineScientific, USA). The slow-freezing process according to the temperature drop gradient set by the computer program followed a protocol previously reported (22–24) using a controlled freezer (PLANER Kryo 360–1.7, UK). The cryotubes were stored in a vapor-phase liquid nitrogen tank at -196°C.

The thawing process for the ovarian tissue began by allowing the cryopreserved samples to thaw at room temperature for approximately thirty seconds. Subsequently, the tissue was immersed in a 37°C water bath for about 2 minutes. Then, it was sequentially transferred into a thawing culture medium with decreasing sucrose concentrations (0.75, 0.375, and 0.125 mmol/L) to remove the cryoprotectant from the ovarian tissue following a protocol previously reported (25). The basic thawing medium contained Dulbecco’s phosphate-buffered saline (Gibco, Grand Island, New York, USA) with added 10% fetal bovine serum.

### 2.3 Animals

Female BALB/C Nude mice, aged 6-7 weeks and weighing approximately 16g, were obtained from Beijing Vital River Laboratory Animal Technology Co., Ltd. The animal license number for the acquisition was SCXK (Beijing, China) 2021-0006. The mice were kept in the SPF laboratory at Capital Medical University. Five mice were housed per cage and provided with pellet food and water ad libitum. They were individually housed in IVC cages under controlled conditions (temperature: 20-22°C, light/dark cycle: 12 hours). Water, food, and bedding were sterilized. The bedding was changed every 2-3 days. The mice underwent a one-week environmental acclimatization period before surgery.

Ovarian tissue xenotransplantation from a single patient was conducted on 1-3 ovariectomized mice, depending on the quantity of ovarian tissue allocated for research. The mice were randomly divided into three groups based on the patient’s MRD status in the bone marrow before cryopreservation: ovariectomized group, subjected to ovariectomized group (n=9); MRD-positive group (n=18); MRD-negative group (n=18).

### 2.4 Surgery

In this study, animal experiments were approved by the Animal Research Committee of Capital Medical University (No: AEEI-2022-182, Date: August 4, 2022). All procedures followed the guidelines for animal welfare in surgical procedures. Surgeries were performed on a sterile heating pad. Anesthesia was induced by intraperitoneal injection of 3% sodium pentobarbital at 45 mg/kg, and the skin was disinfected with iodine. An oblique incision (1cm) was made beneath the rib cage on both sides, cutting through the skin, muscle layer, and peritoneum. Mice in all three groups were ovariectomized. In the MRD-positive group and MRD-negative group, the mice underwent xenotransplantation. Kidneys were gently extruded, exposed, and secured with fine forceps. The tips of fine dissecting forceps were used to make a small incision on the renal capsule. The thawed human ovarian cortex was then processed into 3mm x 3mm pieces, and delicately inserted beneath the renal capsule using flat forceps.

### 2.5 Observation

Animals were closely monitored for death, emaciation, physical condition, and mental status. Body weight was measured weekly. If mice showed signs of emaciation, arched back, depression, and a 20% body mass loss in two weeks, death, severe ascites, or large masses during observation, an immediate record was made. Their livers, spleens, and ovarian grafts were collected for the cause of death analysis.

### 2.6 Tissue collection

When the cryopreserved ovarian tissue was thawed, a portion of the tissue was processed into 4mm x 4mm sections, fixed in 4% paraformaldehyde solution, embedded in paraffin, and then cut into 20 consecutive sections of 3 mm. Every four sections were subjected to hematoxylin and eosin staining (Merck, Darmstadt, Beijing China) for histological evaluation.

Once the mice showed signs of emaciation, arched back, depression, and a 20% body mass loss within two weeks which were mentioned in previously published literature (26, 27) or 6 months post-xenotransplantation, the mice were euthanized using an overdose of sodium pentobarbital to collect blood samples and organ tissues. The general condition of mouse organs, including hepatosplenomegaly, ascites, and the presence of graft masses was observed. Subsequently, livers, spleens, and ovarian grafts were extracted from the mice. The fixation and staining methods were the same as previously described.

### 2.7 Serum hormone assay

Blood samples were collected on the day of graft retrieval after anesthesia, and centrifuged at 3000 rpm to obtain serum, which was then stored at -80°C. Follicle-stimulating hormone (FSH) and estradiol (E2) levels were measured by the FSH enzyme-linked immunosorbent assay (ELISA) Kit (E-EL-M0511c, Elabscience, China) and E2 ELISA kit (E-OSEL-M0008, Elabscience, China).

### 2.8 Viable follicle count in fresh and thawed tissues

1-2 circular ovarian cortical slices processed to a diameter of 2 mm and a thickness of 1 mm were removed from the ovarian tissue randomly for biopsy before the freezing and after the thawing procedure was carried out. The number of viable follicles in the cortical slices was examined by Calcein-AM assay (22, 27).

### 2.9 Pathological examination

Histology and immunohistochemistry were conducted using an Olympus DP controller and manager (Leica, Germany). Morphological detection aligns with prior publications (9, 28). The markers used for immunohistochemistry to evaluate the individual samples of each patient were based on the types of leukemia. Specifically, for acute lymphoblastic leukemia patients, we used CD10, CD79a, CD3, and TdT antibodies. For acute myeloid leukemia patients, we employed CD34, CD117, and MPO antibodies. All assessments were conducted by experienced pathologists.

### 2.10 Statistical Analysis

All data were analyzed by IBM SPSS Statistics 26.0 and graphs were created by GraphPad Prism 8.0. Shapiro-Wilk test was used to assess the normal distribution of the data. The Spearman test was used for analysis of the data on follicle density and age at OTC. For the data on the body weight changes after surgery, Mauchly’s Test of Sphericity was used to determine if there was a correlation between the results at different time points of repeated measurements. For the data on FSH and E2 levels with normal distribution and chi-square variance, one-way ANOVA was used for analysis and Bonferroni *t-test* was used for post-hoc comparisons. Kruskal-Wallis test was used to test for non-normal distribution or heterogeneity of variances. For the data on mortality rate and other survival conditions, the Chi-square test was used for analysis. The data according to normal distribution were represented by “mean ± SEM”. The follicle count data did not demonstrate a normal distribution and were represented by “median and interquartile range”. *p<0.05, **p<0.01, and ***p<0.001 were considered to be significant.

## 3 Results

### 3.1 Viable follicle count in fresh and thawed tissues

**Figure 1:**
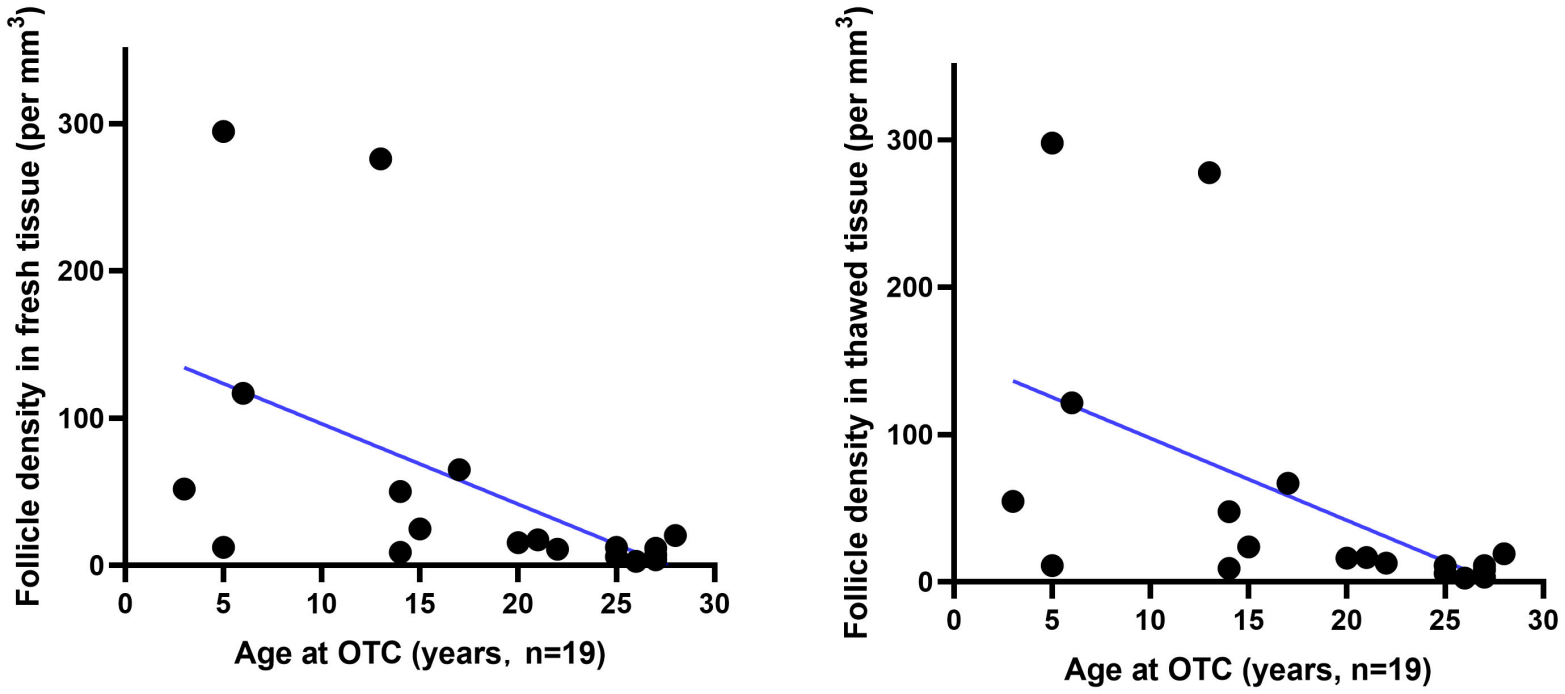
The relationship between follicle density in ovarian tissue and age. Follicle density (per mm^3^) in fresh (n=19) and thawed (n=19) human ovarian tissue declines with age.

The number of viable follicles in fresh tissue with a diameter of 2mm was 49 (28–163) and the number of viable follicles in thawed tissue was 51 (29–142). There was no significant difference (**Table 2**) between the two groups (*p*>0.05). The follicle density in fresh ovarian tissue (**Figure 1** left) was negatively correlated with the patient’s age at OTC (R^2 =^ 0.2906, p=0.0177). The follicle density in the thawed tissue (**Figure 1** right) also showed a similar trend (R^2 =^ 0.2935, *p*=0.0166).

**Table 2:** Viable follicle count in fresh and thawed ovarian tissue.

Group	Number	Viable follicle count	*p*
Fresh tissue	19	49 (28–163)	0.953
Thawed tissue	19	51 (29–172)	

The viable follicle counts in fresh and thawed ovarian tissue. There was no statistically significant difference in viable follicular counts between the two groups, *p*>0.05.

### 3.2 The changes in body weight in each group after surgery

**Figure 2:**
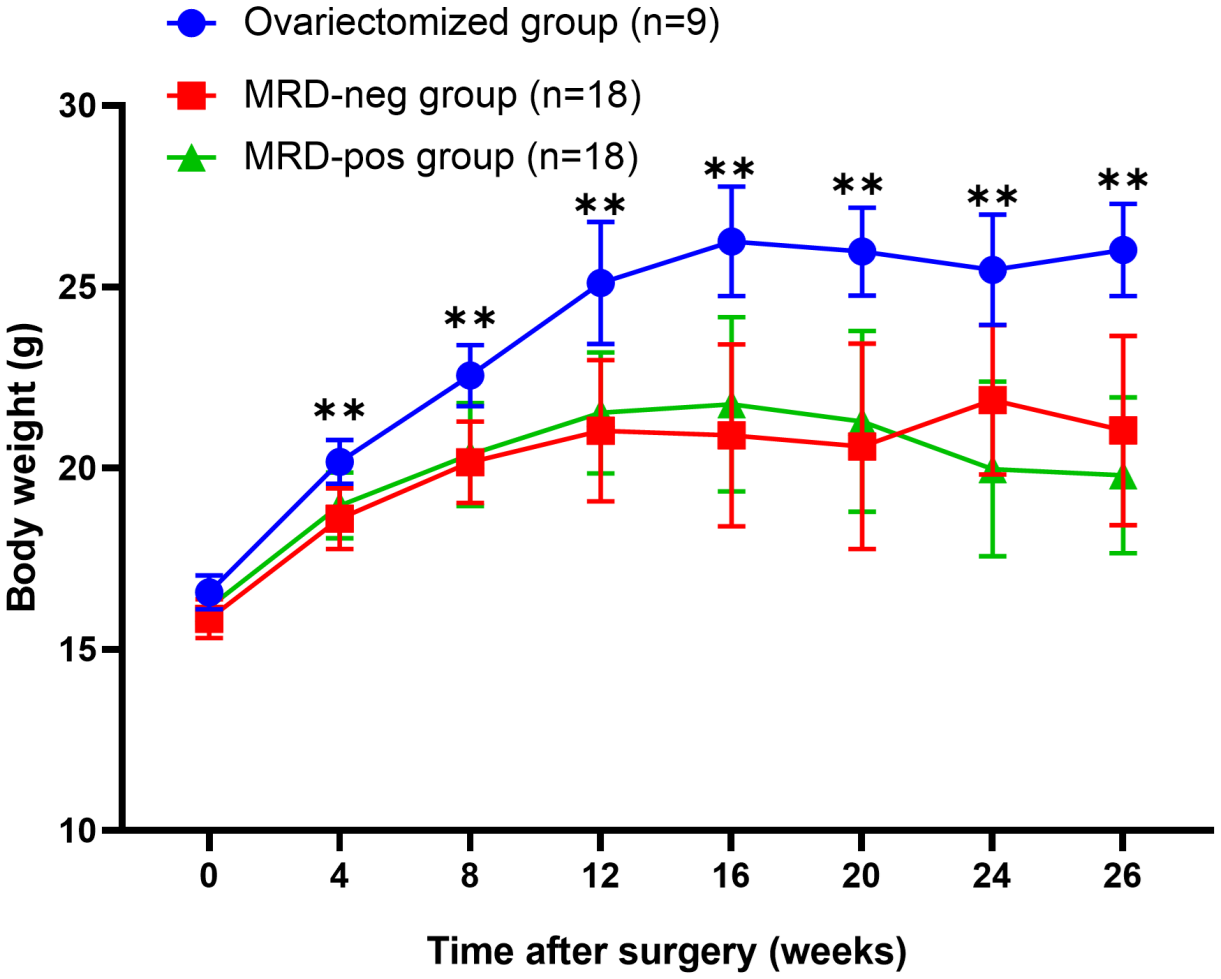
The body weight of the mice after surgery showed that the body weight of the ovariectomized group (n=9) was significantly higher than that of the MRD-negative (n=18) and MRD-positive (n=18) groups. ** *p*<0.01.

At baseline state, no statistical difference (*p* > 0.05) was observed in body weight between the ovariectomized group (16.23 ± 1.25 g) and both the MRD-negative group (16.01 ± 0.65 g) and the MRD-positive group (16.12 ± 0.49 g). However, from 4 weeks to 26 weeks after surgery, the ovariectomized group exhibited a significantly higher weight compared to the other two groups (*p* < 0.01). There was no significant difference in body weight between the MRD-negative and MRD-positive groups (*p* > 0.05). The changes in body weight after xenotransplantation were illustrated in **Figure 2**.

### 3.3 Hormonal assays

**Figure 3:**
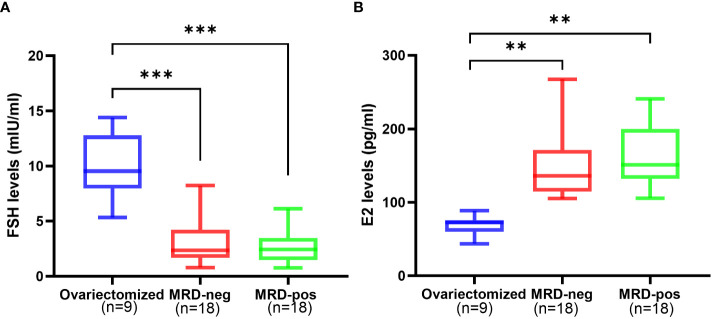
The hormone levels after 6 months of xenotransplantation showed that FSH levels **(A)** were significantly higher in the ovariectomized group (n=9) compared to the MRD-negative group (n=18, ^***^ *p*<0.001)and MRD-positive group (n=18, ^***^ *p*<0.001), and E2 **(B)** levels were significantly lower compared to the MRD-negative group(n=18, ^**^ *p*<0.01)and MRD-positive group (n=18, ^**^ *p*<0.01). However, there were no statistical differences in FSH and E2 levels between the MRD-negative and MRD-positive groups. FSH: follicle stimulating hormone (mIU/ml); E2: estradiol (pg/mL).

FSH level showed no significant difference in the MRD-negative group (2.74 ± 1.58 mIU/ml) compared to the MRD-positive group (3.04 ± 1.91 mIU/ml, *p* > 0.05), but both of the two groups exhibited a significantly lower FSH level compared to the ovariectomized group (9.92 ± 2.89 mIU/ml) (*p*<0.001, **Figure 3A**). There was no significant difference in E2 level between the MRD-negative group (162.23 ± 38.27 pg/ml) and the MRD-positive group (163.52 ± 84.72 pg/ml) (p > 0.05). However, both groups showed significantly higher E2 levels compared to the ovariectomized group (68.90 ± 13.34 pg/ml, *p <*0.01, **Figure 3B**).

### 3.4 Abnormal conditions observed after xenotransplantation

30 of the 45 mice survived to the end of the observation. None of the mice in the MRD-negative group (n=18) showed abnormal conditions such as death, emaciation, or hepatosplenomegaly during the observation period. Except one of them (1/18) was euthanized for ascites 16 weeks after xenotransplantation. On autopsy, follicles were visible in the ovarian grafts, no mass was seen, and the liver and spleen were not enlarged. Ascites sample and organ tissue were examined microscopically, and the cause of ascites in the mice was assessed to be unrelated to leukemic cell contamination. 14 mice in the MRD-positive group (n=18) died during the observation period, and 10 mice had emaciation, 3 mice had hepatosplenomegaly and graft mass.

**Figure 4:**
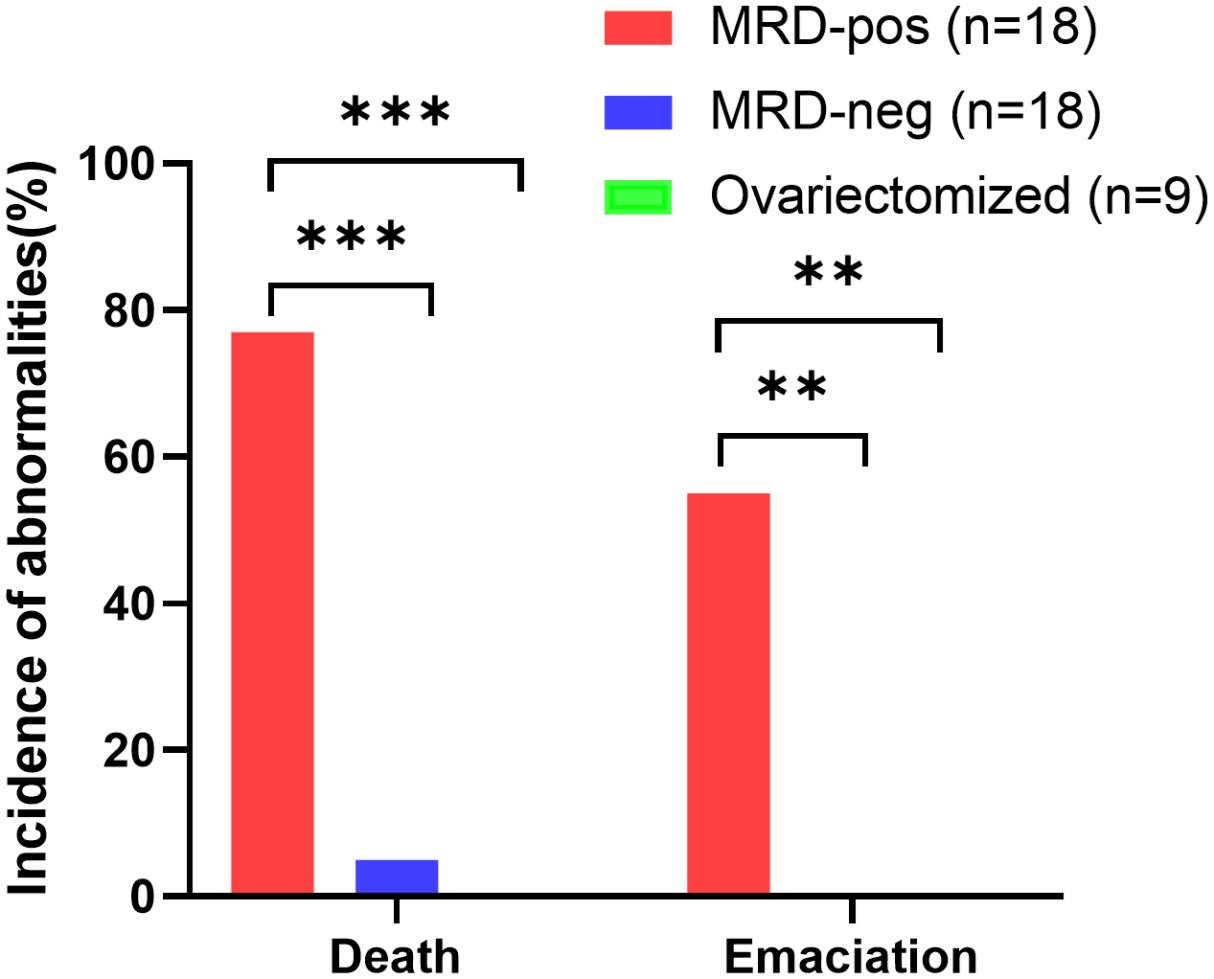
Quantification of the proportion of animals with abnormalities (death and emaciation) in the MRD-positive group (n=18, red bars), MRD-negative group (n=18, blue bars), and ovariectomized group (n=9, green bars), ***p<0.001, **p<0.01.

The MRD-positive group showed a more frequent mortality rate (*p*<0.001), and emaciation (*p*<0.01), compared with the MRD-negative group and ovariectomized group (**Table 3**, **Figure 4**). Referred to other abnormal conditions such as hepatosplenomegaly, ascites, and graft mass (**Table 3**), there was no statistical significance between the two groups. Abnormal conditions observed after xenotransplantation were shown in **Table 3**.

**Table 3:** Abnormalities observed after xenotransplantation into mice.

Group	Number	Death (n)	Emaciation (n)	Ascites (n)	Hepatosplenomegaly (n)	Graft abnormality (n)
MRD-pos	18	14(77.8%)	10(55.6%)	4(21%)	3(16.7%)	3(16.7%)
MRD-neg	18	1(5.6%)	0(0%)	1(5.3%)	0(0%)	0(0%)
ovariectomized	9	0(0%)	0%	0(0%)	0(0%)	0(0%)
*P-value*		<0.001***	<0.01**	0.17	0.114	0.114

MRD-neg: MRD-negative group, MRD-pos: MRD-positive group, ovariectomized: ovariectomized group, **p < 0.01, ***p<0.001.

### 3.5 Macroscopic observations

#### 3.5.1 Reproductive organs

**Figure 5:**
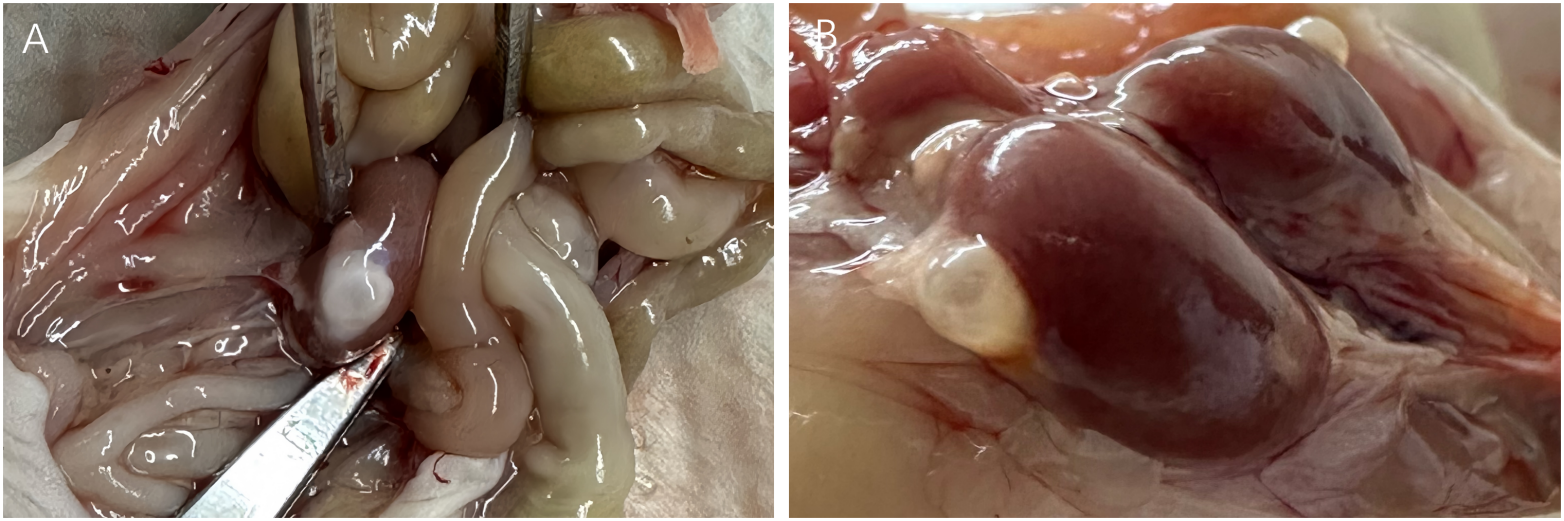
26-weeks post-xenotransplantation, antral follicles (>2mm in diameter) were observed in grafts on the kidney surface of the mice (**A**: Patient 18 and **B**: Patient 3).

**Figure 6:**
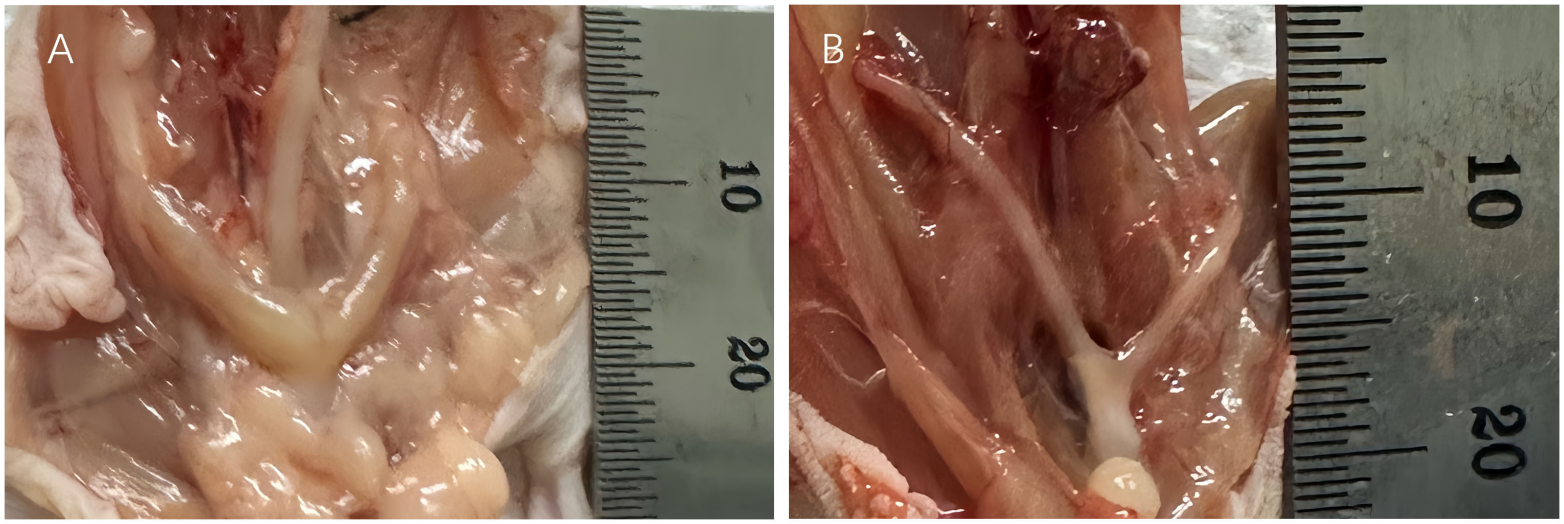
The uterus of the xenotransplanted mice (**A**: Patient 15) was bigger than the urine of the ovariectomized mice **(B)**.

After 26 weeks of xenotransplantation, the volume of the ovarian grafts became smaller, but all of them could be identified and the follicle growth was visual when removed (**Figure 5**). However, 3 mice (Patient 9) in the MRD-positive group showed ovarian graft mass (**Table 3**). Moreover, the uterus volume was bigger in the MRD-negative and MRD-positive groups, compared with the ovariectomized group (**Figure 6**).

#### 3.5.2 Liver and spleen

**Figure 7:**
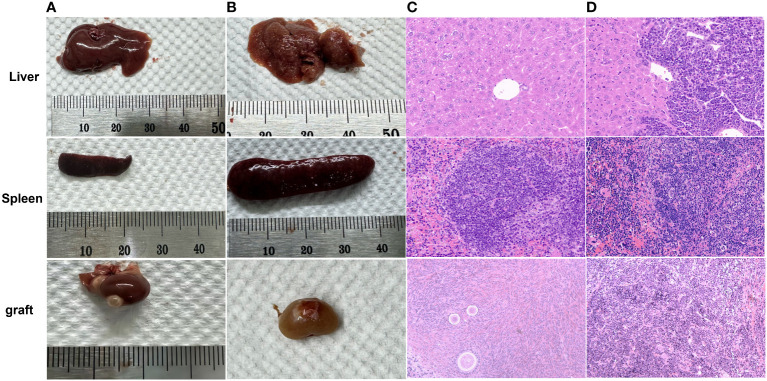
Macroscopic observation of normal **(A)** and abnormal **(B)** livers, spleens, and grafts. Leukemic cell infiltration was seen in the livers, spleens, and grafts of 3 abnormal mice (Patient 9) in the MRD-positive group **(D)** compared with those **(C)** of mice (Patient 18) with morphological normality under the microscope (HE staining, 40×).

All mice in the MRD-negative group showed no significant abnormalities in the appearance of the livers and spleens. 3 mice (Patient 9) in the MRD-positive group showed abnormalities in the macroscopic appearance of the livers and spleens (**Figure 7**, **Table 3**).

### 3.6 Histological evaluations

#### 3.6.1 Thawed ovarian tissue

A 2mm*2mm*1mm ovarian cortical tissue was obtained when the cryopreserved ovarian tissue was thawed. All samples exhibited follicles, and no malignant cells were detected by histology observation or immunohistochemistry.

#### 3.6.2 Ovarian grafts

**Figure 8:**
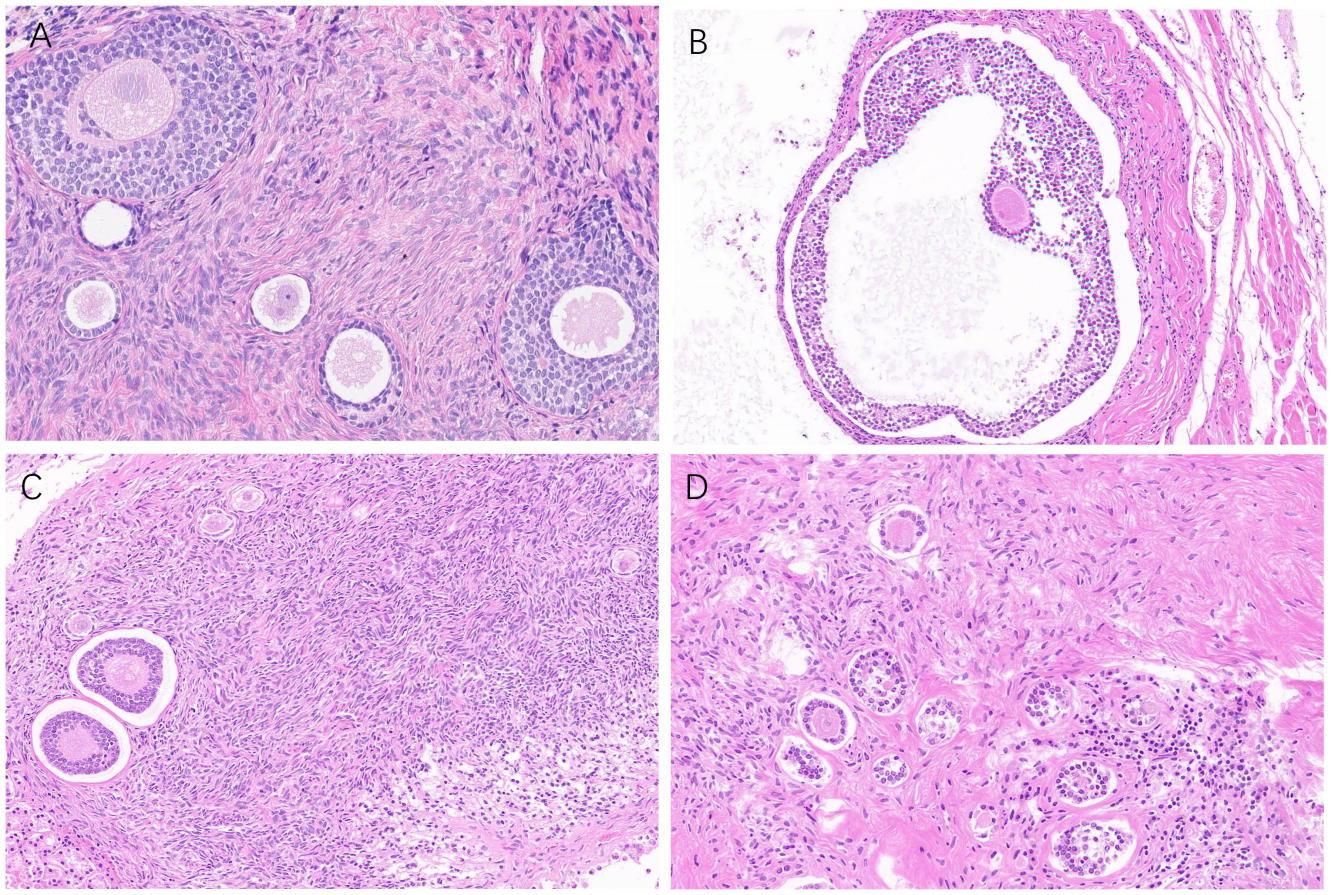
Follicle growth (**A**: Patient 18, **B**: Patient 3, **C**: Patient 13, and **D**: Patient 5) was observed in ovarian grafts removed 26 weeks after xenotransplantation (HE staining, 40 ×).

All ovarian grafts were removed 6 months after xenotransplantation in the MRD-negative group of mice. As shown in **Figure 8**, serial sections showed normal human ovarian histology with different grades of follicle growth. No malignant cells were detected by immunohistochemistry or histology observation. However, three of the ovarian grafts removed from the MRD-positive group of mice (Patient 9) showed significant lymphocytic invasion (**Figure 7D**).

#### 3.6.3 Liver and spleen of mice

Compared with the livers and spleens of normal mice (**Figures 7A, B**), the livers and spleens of three nude mice (whose ovarian grafts showed significant lymphocytic invasion) with enlarged livers and spleens (Patient 9) in the MRD-positive group were also infiltrated with leukemia cells, and the cells grew in a clumping pattern and formed nodules (**Figures 7C, D**). The spleen had hyperplasia of red and white marrow with blurred boundaries and the structure of the liver was necrotic.

## 4 Discussion

Our research has demonstrated that after xenotransplantation of ovarian tissue from leukemia patients, the ovarian grafts not only survived but also facilitated follicular development and maintained ovarian endocrine function. Additionally, we observed a lower risk of malignant cell re-engraftment in leukemia patients with MRD negativity in the bone marrow before OTC.

Despite leukemia being considered high-risk for ovarian tissue autotransplantation (29), this remains the primary method for preserving fertility in leukemia patients. Therefore, the safety of autotransplantation in leukemia patients is a great concern for both doctors and patients.

Using molecular biology techniques such as RQ-PCR, fluorescence *in situ* hybridization (FISH), NGS, and MFC to search for evidence of MRD in ovarian tissue is a current focus in the safety studies of OTT (11, 27, 30). Nevertheless, the outcomes of MRD detection are constrained by the sensitivity of the detection method. A positive MRD result suggests the potential presence of malignant cells in ovarian tissue. However, whether these malignant cells can cause tumor recurrence is yet to be determined. The immunodeficient mice xenograft model as an alternative to the human bio incubator is a sensitive method to evaluate whether malignant cells can survive *in vivo (* 31–33). Dolmans (9) used histology, RQ-PCR, and xenotransplantation to assess the presence of leukemia cells in the ovarian tissue of 18 patients with acute and chronic leukemia. Seven cases tested positive at the molecular level, and four exhibited macroscopic masses. This led to the conclusion that OTT was unsafe in leukemia patients. However, the vast majority of cases with malignant cell contamination in the ovaries were not undergoing treatment for the primary disease at the time of OTC, and only two cases received a full cycle of intravenous chemotherapy before OTC. With the development of leukemia diagnostic and treatment techniques, most patients have chemotherapy before OTC. The risk of malignant cell contamination in the ovarian tissue of leukemia patients may vary based on the disease status. Jahnukainen used RQ-PCR assay to examine the presence of malignant cell contamination in ovarian tissue with different bone marrow MRD status, finding an association between MRD status in bone marrow at the time of OTC and MRD status in ovarian tissue (12). However, evidence of malignant cell contamination at the molecular level does not definitively determine whether transplantation will necessarily lead to disease recurrence (31). Further, *in vivo* experiments are still needed to validate the survival capability of malignant cells. Our study is the first *in vivo* study to use xenotransplantation, comparing the risk of disease induction in OTT from acute leukemia patients with different bone marrow MRD statuses.

Our research team has conducted multiple studies previously (23, 24, 34), all of which have evaluated and demonstrated that there are no significant differences in follicular activity between fresh and thawed ovarian tissue. Furthermore, we have established an immunodeficient mice xenotransplantation model previously (35), confirming the survival of follicles within ovarian grafts post-xenotransplantation. This study is built upon these foundations and focuses on investigating the safety and efficacy of ovarian tissue transplantation. Due to the fact that some of these ovarian tissues were sourced from prepubertal girls, the ovarian tissue obtained in this study were limited and precious. Our sampling is conducted randomly, and each patient’s ovarian tissue is transplanted into multiple mice, ensuring that the sampled sites are representative and can reflect the overall situation. Our study confirms that after ovariectomy and subsequent transplantation of human ovarian tissue, the mice exhibit restored ovarian endocrine function. The selection of the renal capsule as the transplant site is due to its rich blood supply, facilitating the vascularization of the graft, and its distinct anatomical location, making it easily identifiable during recovery (31, 33). Over the 26-week observation period post-surgery, the mice in the ovariectomized group experienced an increase in body weight, akin to the weight gain observed in postmenopausal women due to ovarian functional depletion, leading to disruptions in glucose and lipid metabolism (36, 37). In contrast, ovariectomized mice xenotransplanted with human ovarian tissue showed significantly lighter body weights, indicating that ovarian grafts exert a beneficial effect on metabolism. Macroscopically and microscopically, ovarian grafts exhibited various levels of follicles, morphologically demonstrating the survival and development of ovarian grafts after heterotopic transplantation. Additionally, compared to mice in the ovariectomized group, those undergoing xenotransplantation displayed significantly lower serum FSH levels and higher E2 levels, indicating the endocrine functionality of the ovarian grafts. Considering serum hormone levels, follicle morphology, and the impact on mouse metabolism, we successfully established a long-term xenotransplantation model of cryopreserved ovarian tissue from leukemia patients, laying the foundation for using mice as an animal model to replace human subjects in safety assays.

This study found that leukemia patients with MRD-negative bone marrow before OTC have a lower risk of malignant cell reimplantation. During the postoperative observation period, mice in the MRD-negative group showed no signs of death, emaciation, hepatosplenomegaly, or other abnormalities, both macroscopically and histologically. In contrast, mice in the MRD-positive group exhibited a significantly higher incidence of death and emaciation during the observation period. Although there was no statistical difference in hepatosplenomegaly and ascites between the two groups, the small sample size prevents a conclusive clinical interpretation. Histological examinations further demonstrated leukemia cell infiltration in mice with hepatosplenomegaly, indicating infiltration of leukemia cells into the grafts. The results observed in our xenotransplantation model align with the findings of Jahnukainen’s team, who detected MRD-negative leukemia patients’ ovarian tissue at the molecular level (12). Chemotherapy appears to eliminate leukemia cell contamination in ovarian tissue to some extent. This suggests a lower risk of malignant cell contamination in ovarian tissue from leukemia patients with bone marrow MRD negativity at the time of cryopreservation, making autotransplantation safer. Therefore, it is essential to reassess the risk of ovarian tissue transplantation in MRD-negative patients. Moreover, for patients with recurrent MRD positivity after chemotherapy, recognizing the importance of transplantation safety evaluation is crucial, emphasizing the need for caution in autotransplantation. The important clinical value of this study lies in providing recommendations for the timing selection of ovarian tissue cryopreservation in leukemia patients. For leukemia patients who were MRD-positive in bone marrow before OTC, there is a higher risk of malignant cell engraftment during autotransplantation. It is advisable to strive for patients to achieve bone marrow MRD negativity after treatment before undergoing OTC, thereby enhancing the safety of autotransplantation.

Chemotherapy may impact ovarian function in leukemia patients, and the severity is closely related to the type of chemotherapy drugs used (38). Alkylating agents such as cyclophosphamide and busulfan can directly damage the follicle pool, cause vascular injury, and lead to ovarian fibrosis (39, 40). Most leukemia patients undergo several rounds of chemotherapy after diagnosis to achieve optimal treatment results—MRD-negative in bone marrow. However, the drugs commonly used for induction chemotherapy generally do not contain high doses of alkylating agents (41). As long as myeloablative conditioning (commonly using high doses of alkylating agents) before HSCT is not performed, the patient’s ovarian function is not likely to be severely damaged (42). The European Society of Human Reproduction and Embryology (ESHRE) recommends that “patients who have undergone low gonadotoxic treatment can choose ovarian tissue cryopreservation to preserve fertility” (4). Therefore, we highly recommend leukemia patients undergo OTC when achieving MRD-negative in bone marrow to reduce the risk of malignant cell reimplantation.

The results of MRD status are determined by the quantity of tissue samples and the sensitivity threshold of the detection method (15). Currently, no method exists to determine the minimum number of malignant cells that can trigger leukemia (28). Therefore, there is no guarantee of the safety of OTT in leukemia patients. For leukemia patients who undergo OTT after safety evaluation indicates a low risk of malignant cell contamination, it is essential to conduct long-term and close follow-ups.

This study has several limitations. Since the age range of patients involved in this study varied significantly, we did not conduct graded follicle counting but rather counted total follicles. In future studies, we will consider age matching to describe the follicle growth in more detail. Due to the lower sensitivity of the xenotransplantation compared to the molecular assays, combining high-sensitivity methods such as RQ-PCR, MFC, FISH, etc., simultaneously would provide a more comprehensive and convincing assessment of the safety of autotransplantation of cryopreserved ovarian tissue from both microscopic and macroscopic perspectives. Additionally, expanding the sample size and further subdividing based on different leukemia disease states into four groups—CR with MRD negativity, CR with MRD-positive, partial remission, and none remission—would enable a more thorough understanding of the risk variations in patients undergoing OTT in different disease conditions. This approach would contribute to a more individualized formulation of fertility preservation plans for patients.

Thus, OTC in acute leukemia patients with MRD-negative bone marrow appears to be a safe approach. Further research efforts should focus on exploring methods to eliminate malignant cells from the ovarian tissue of leukemia patients and alternative approaches, such as artificial ovaries, *in vitro* activation of primordial follicles, and *in vitro* growth and maturation of primordial follicles. These developments aim to provide more and safer options for fertility preservation in leukemia patients.

## 5 Conclusion

As the first and the only centralized human ovarian tissue cryobank in China (23, 34), it is essential to demonstrate reliable testing within the new bank. What’s more, the data on ovarian tissue cryopreservation and transplantation in leukemia patients are extremely scarce globally and represent a hot topic. This study conducted long-term xenotransplantation with a relatively large sample size, making it valuable for evaluating the safety and efficacy of ovarian tissue cryopreservation and transplantation in leukemia patients. Our study showed the efficacy and safety of cryopreserved ovarian tissue transplantation from acute leukemia patients via xenotransplantation. We observed that the grafts not only survived but also promoted follicle development and maintained ovarian endocrine function. Additionally, we noted a lower risk of malignant cell reimplantation in leukemia patients with MRD-negative bone marrow before ovarian tissue cryopreservation. Therefore, we recommend leukemia patients strive to achieve MRD-negative bone marrow before undergoing OTC.

## Data availability statement

The original contributions presented in the study are included in the article/supplementary material. Further inquiries can be directed to the corresponding author.

## Ethics statement

The studies involving humans were approved by the Ethics Committee of Beijing Obstetrics and Gynecology Hospital, Capital Medical University (ethics NO: 2020-KY-007-01). The studies were conducted in accordance with the local legislation and institutional requirements. Written informed consent for participation in this study was provided by the participants’ legal guardians/next of kin. The animal study was approved by Ethics Committee on Animal Research of the Capital Medical University (ethics NO: AEEI-2022-182). The study was conducted in accordance with the local legislation and institutional requirements.

## Author contributions

YL: Data curation, Formal Analysis, Investigation, Software, Writing – original draft, Writing – review & editing. XR: Writing – review & editing. MG: Investigation, Writing – review & editing. JD: Writing – review & editing. FJ: Writing – review & editing. JC: Investigation, Writing – review & editing. YL: Writing – review & editing. LJ: Investigation, Writing – review & editing. ZW: Writing – review & editing. YY: Investigation, Writing – review & editing. MZ: Investigation, Writing – review & editing. AM: Writing – review & editing.
